# AI diagnostic performance based on multiple imaging modalities for ovarian tumor: A systematic review and meta-analysis

**DOI:** 10.3389/fonc.2023.1133491

**Published:** 2023-04-21

**Authors:** Lin Ma, Liqiong Huang, Yan Chen, Lei Zhang, Dunli Nie, Wenjing He, Xiaoxue Qi

**Affiliations:** ^1^Department of Obstetrics and Gynecology, Chengdu First People's Hospital, Chengdu, China; ^2^Department of Ultrasound, Chengdu First People's Hospital, Chengdu, Chengdu, China; ^3^Big Data Research Center, University of Electronic Science and Technology of China, Chengdu, China

**Keywords:** ovarian cancer, AI, ultrasound, meta-analysis, systematic review

## Abstract

**Background:**

In recent years, AI has been applied to disease diagnosis in many medical and engineering researches. We aimed to explore the diagnostic performance of the models based on different imaging modalities for ovarian cancer.

**Methods:**

PubMed, EMBASE, Web of Science, and Wanfang Database were searched. The search scope was all published Chinese and English literatures about AI diagnosis of benign and malignant ovarian tumors. The literature was screened and data extracted according to inclusion and exclusion criteria. Quadas-2 was used to evaluate the quality of the included literature, STATA 17.0. was used for statistical analysis, and forest plots and funnel plots were drawn to visualize the study results.

**Results:**

A total of 11 studies were included, 3 of them were modeled based on ultrasound, 6 based on MRI, and 2 based on CT. The pooled AUROCs of studies based on ultrasound, MRI and CT were 0.94 (95% CI 0.88-1.00), 0.82 (95% CI 0.71-0.93) and 0.82 (95% Cl 0.78-0.86), respectively. The values of I^2^ were 99.92%, 99.91% and 92.64% based on ultrasound, MRI and CT. Funnel plot suggested no publication bias.

**Conclusion:**

The models based on ultrasound have the best performance in diagnostic of ovarian cancer.

## Introduction

Ovarian cancer (OC) is the malignant tumor with the highest death rate in the female reproductive system, with a high incidence rate and mortality (1). Among gynecological tumors, the incidence rate ranks third and the mortality ranks first, surpassing cervical cancer and endometrial cancer, posing a serious threat to the health of women (2). Unnecessary surgery leads to reduced fertility, therefore, accurate preoperative assessment of the risk of malignancy can help physicians provide individualized treatment for patients (3).

At present, the diagnosis of OC mainly relies on pathological examination and medical imaging techniques which can assist in the diagnosis and treatment of OC (4). However, due to the insidious nature of OC, the physician’s visual inspection of medical images can’t provide enough information to personalize the treatment for the patient (5). Artificial intelligence (AI) can automatically recognize complex patterns in imaging data, extract potential information from medical images, and provide quantitative assessment of radiographic characteristics (6). The application of artificial intelligence in medical imaging mainly includes two categories of radiomics and deep learning (7). This non-invasive approach reduces patient pain and helps physicians personalize treatment for patients.

For OC patients, proper and accurate preoperative imaging is very important for the treatment of cancer. Ultrasound is mainly used for early screening of ovarian cancer (8). The computed tomography (CT) imaging is the standard for preoperative evaluation of patients with OC; magnetic resonance imaging (MRI) focuses on imaging small peritoneal deposits in difficult-to-resect areas (9). To our knowledge, a recent study conducted Mata-analysis of studies which early predicted different kinds of diseases based on AI, demonstrating the important role of AI in disease diagnosis (10). Another study evaluated the diagnostic performance of artificial intelligence for lymph node metastasis in abdominopelvic malignancies and found that the diagnostic ability of artificial intelligence was higher than the subjective judgment of physicians (11). However, these studies have ignored the impact of different imaging modalities on artificial intelligence diagnostic results.

The purpose of this study was to conduct a systematic review and meta-analysis of published data on ovarian cancer to assess the accuracy of artificial intelligence in the application of multiple imaging modalities for OC.

## Methods

### Search strategy

In this study, the Preferred Reporting Item of the Guidelines for Systematic Reviews and Meta-Analysis (PRISMA) was used as the search rule (12), and the databases used for the search were PubMed, EMBASE, Web of Science, and Wanfang Database. **Table 1** shows the method of search. The search was conducted using subject terms including “radiomics,” “deep learning,” “Artificial intelligence,” “ovarian cancer,” and “malignant ovarian tumors”. Combine the results of different queries by using the Boolean operator AND. Any eligible studies were considered preliminary search results. To get all relevant literatures, we searched the reference list of relevant studies by manual search.

**Table 1:** Search Strategy.

Sources	Search in	MeSH terms	Limits	Search results
Web of Science	Search manager	(“Artificial intelligence” OR”AI” OR”deep learning” OR “machine learning” OR “radiomics” OR “radiomic”) AND (“CT” OR “MRI”OR “ultrasound”) AND (“ovarian cancer” OR “ malignant ovarian tumors” OR “ OC “)	None	5
PubMed, (MEDLINE)	N/A	(“Artificial intelligence” OR”AI” OR”deep learning” OR “machine learning” OR “radiomics” OR “radiomic”) AND (“CT” OR “MRI”OR “ultrasound”) AND (“ovarian tumors” OR “benign and malignant ovarian tumors”) AND (“ovarian cancer” OR “ OC “)	None	29
EMBASE	Quick search	(‘Artificial intelligence’/exp OR ‘Artificial intelligence’ OR ‘AI’/exp OR ‘AI’OR ‘machine learning’/exp OR ‘machine learning’ OR ‘radiomics’/exp OR ‘radiomics’ OR ‘radiomic’) AND (‘ct’/exp OR ‘ct’ OR ‘mri’/exp OR ‘mri’OR ‘ultrasound’/exp OR ‘ultrasound’) AND (‘ovarian tumors’/exp OR ‘ovarian tumors’ OR ‘benign and malignant ovarian tumors’/exp OR ‘benign and malignant ovarian tumors’) AND (‘ovarian cancer’/exp OR ‘ OC ‘)	None	30
Wanfang Database	N/A	(“Artificial intelligence” OR”AI” OR”deep learning” OR “machine learning” OR “radiomics” OR “radiomic”) AND (“CT” OR “MRI”OR “ultrasound”) AND (“ovarian tumors” OR “malignant ovarian tumors”) AND (“ovarian cancer” OR “ OC “)	None	7

N/A, Not Applicable.

### Study selection

The inclusion and exclusion criteria for studies were as follows. Inclusion criteria: (1) retrospective or prospective studies evaluating the diagnostic efficacy of AI in identifying ovarian tumors of patients; and (2) patients with ovarian tumor. Exclusion criteria: (1) animal studies, case reports, conference literature; (2) insufficient computable data; and (3) duplicate reports or studies based on the same data. Two researchers used Covidence software to screen studies and identified titles and abstracts. Disagreements in the process of study screening were arbitrated and agreed upon by a third author.

### Data extraction

Data were extracted from all eligible studies, and information extracted included:first author, country, year of publication, type of AI model, number of patients, age of patients, type of tumor, type of stu1dy and imaging modality. The area under the receiver operating characteristic curve (AUROC), sensitivity (SEN), specificity (SPE), and accuracy are used to evaluate the performance of the models, with AUROC being considered the most important metric. The data we extracted was used for data processing and forest map production.

### Quality assessment

The Quality Assessment of Diagnostic Accuracy Studies Scale (QUADAS-2) was used to assess the risk of bias of included studies (13). First, the two researchers responded to each study’s landmark questions using three options: “yes”, “no”, and “uncertain”. Then the third researcher used the QUADAS-2 to rate the risk of bias into three categories, “low,” “high,” or “uncertain”.

### Statistical analysis

Meta-analysis of the included literature was implemented in this study using STATA 17.0. If the study population is divided into training and test sets, only the test set data are included as metrics. If multiple models were used simultaneously in a given study, we only select the model with the median AUROC value. Continuous variables were described using mean difference (MD) as well as 95% confidence interval (CI), and were considered statistically significant when P<0.05. Heterogeneity was assessed according to discordance index (I^2^) (14). If I^2^<50%, it indicated low heterogeneity of Meta-analysis results and a fixed-effect model could be selected. Contrary, if I^2^≧50% that indicated high heterogeneity of Meta-analysis results and a random-effect model could be selected. Funnel plots and Egger tests (15) were used to assess whether there was publication bias in the results of Meta-analysis. When publication bias existed, the results of Meta-analysis were further analyzed for stability and reliability using the cut-and-patch method. In addition, sensitivity analysis was used to assess the robustness of the results of Meta-analysis. Sensitivity analyses excluding one study at a time were conducted to clarify whether the results were driven by one large study or a study with an extreme result.

## Results

### Study selection

**Figure 1:**
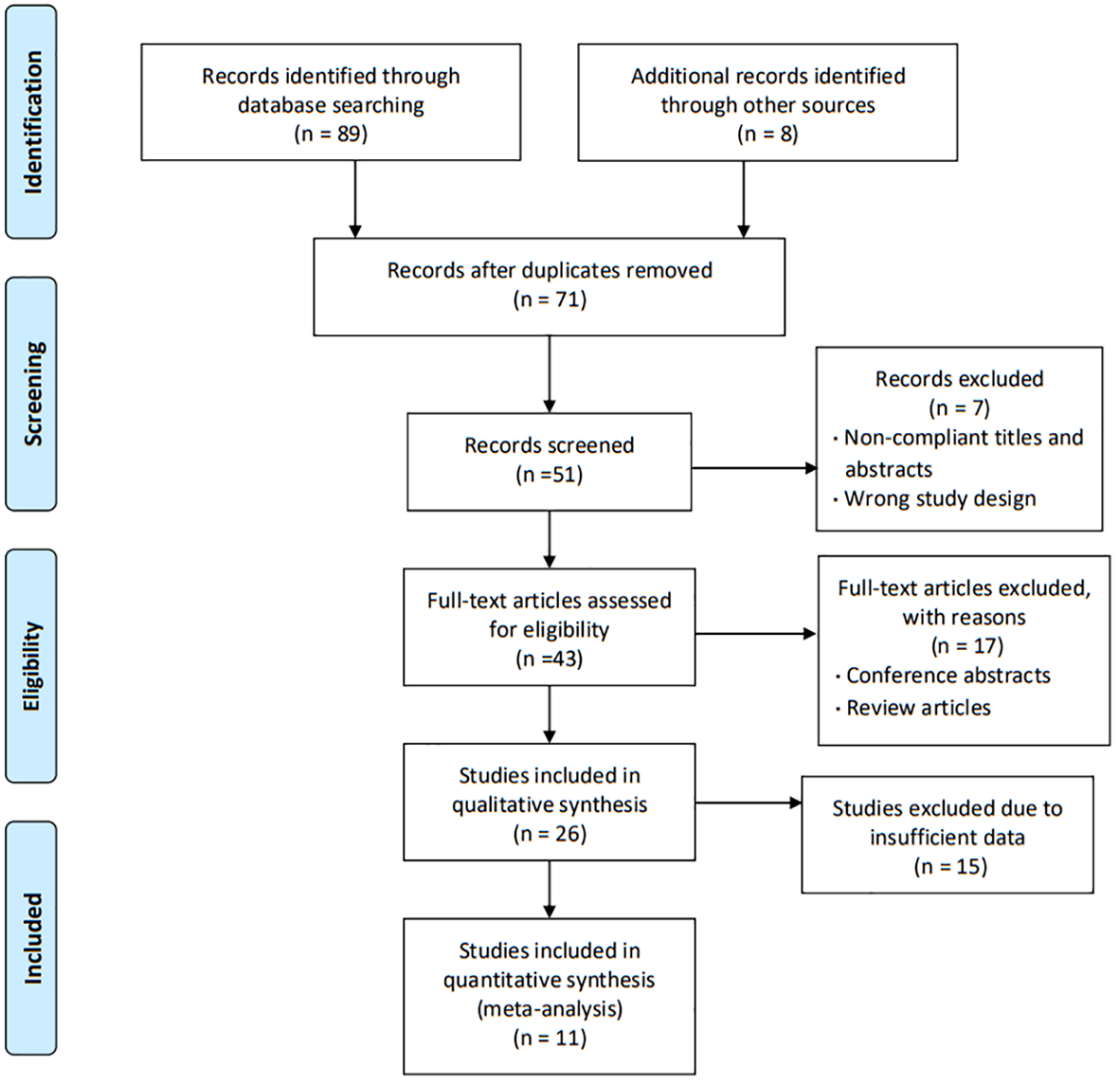
Study selection process.

In total, 71 studies were identified after removing duplicates, but 20 studies with non-compliant titles and abstracts were excluded. After full-text screening of the remaining 51 studies, only 26 studies met the requirements, but 15 of them had insufficient data, and the last 11 studies were used in our Meta-analysis (16–25). **Figure 1** shows the selection process of our study.

### Study characteristics

**Figure 2:**
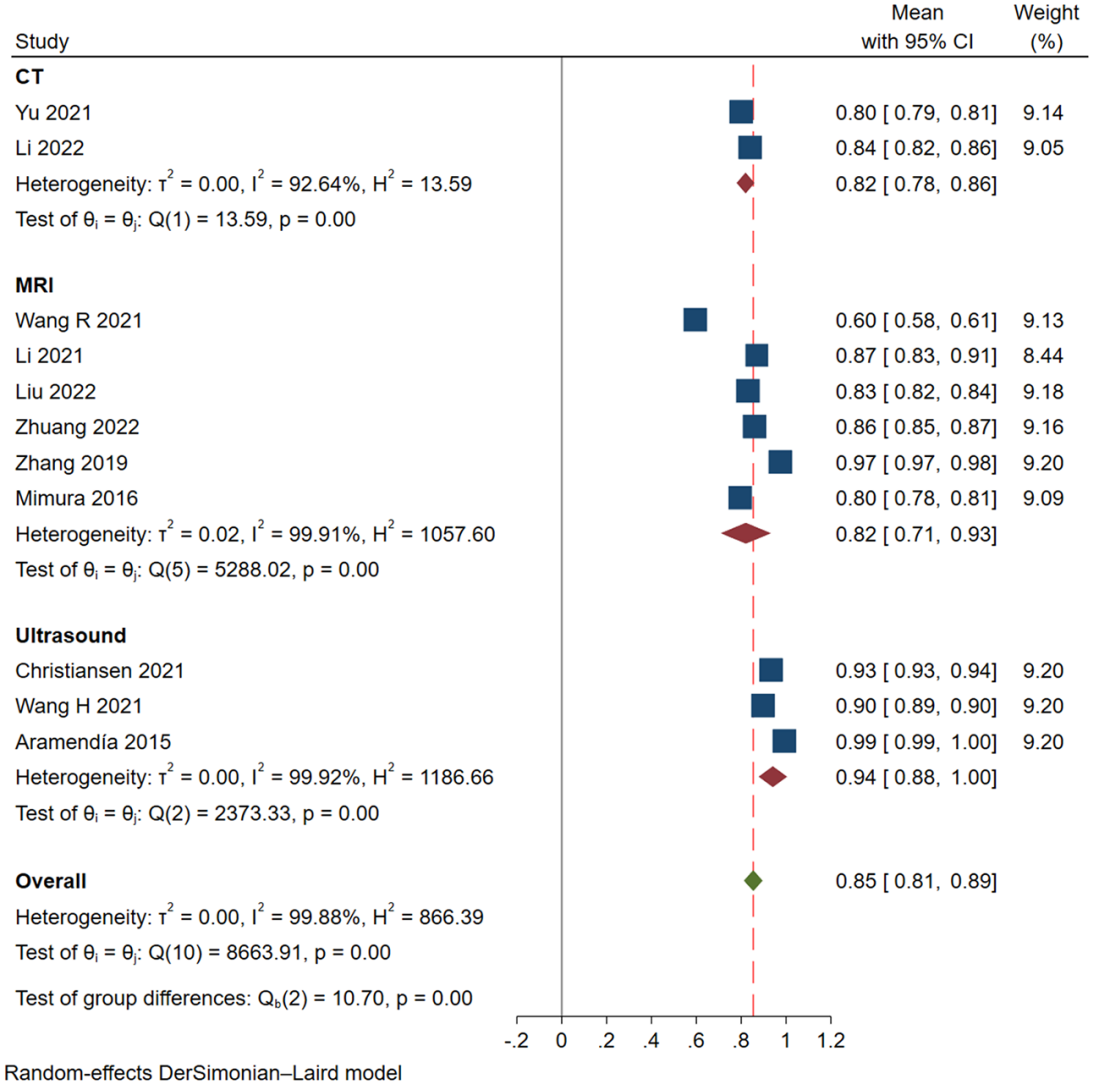
Forest plots of Meta-analysis.

We finally selected 11 studies for meta-analysis, and the characteristics of each study are summarized in **Table 2**. All of the studies we screened were retrospective, and two of them had independent validation set. Four studies built deep learning models and seven built radiomics models. In addition, the gold standard of diagnosis in most studies is pathology. In these studies, 3 types of medical imaging were used, 3 with ultrasound, 6 with MRI, and only 2 with CT. The results of the Meta-analysis of the AUROC values are presented in the form of forest plots in **Figure 2**.

**Table 2:** Selected characteristics.

First Author	Country	Year	Study design	Patients	Mean or Median age (SD; range), years	Imaging modality	Type of malignancy	AI model (Per-patient/per-node diagnostic output)	Reference standard
Christiansen-1	Sweden	2021	Retrospective Single-center	758	_	Ultrasound	Ovarian tumors	Deep learning (per-patient)	Pathology
Wang-2	China	2021	Retrospective Single-center	265	_	Ultrasound	Ovarian tumors	Deep learning (per-patient)	Pathology
Aramendía-Vidaurreta-3	Spain	2015	Retrospective Single-center	145	43(35-65)	Ultrasound	Ovarian tumors	Deep learning (per-patient)	_
Wang-4	USA	2021	Retrospective Single-center	451	47.8	MRI	Ovarian tumors	Deep learning (per-patient)	Pathology
Li-5	China	2021	Retrospective Multi-center	134	47.3	MRI	Ovarian tumors	Radiomics (per-patient)	Radiology
Liu-6	China	2022	Retrospective Single-center	196	45.85(13.5)	MRI	Ovarian tumors	Radiomics (per-patient)	Pathology
Zhuang-7	China	2022	Retrospective Single-center	91	37	MRI	Ovarian tumors	Radiomics (per-patient)	Pathology
Zhang-8	China	2019	Retrospective Multi-center	286	_	MRI	Ovarian tumors	Radiomics (per-patient)	Pathology
Mimura-9	Japan	2016	Retrospective Single-center	42	49.7	MRI	Ovarian tumors	Radiomics (per-patient)	Pathology
Yu-10	China	2021	Retrospective Single-center	182	47.1	CT	Ovarian tumors	Radiomics (per-patient)	Pathology
Li-11	China	2022	Retrospective Single-center	140	_	CT	Ovarian tumors	Radiomics (per-patient)	Pathology

### Quality assessment

**Figure 3:**
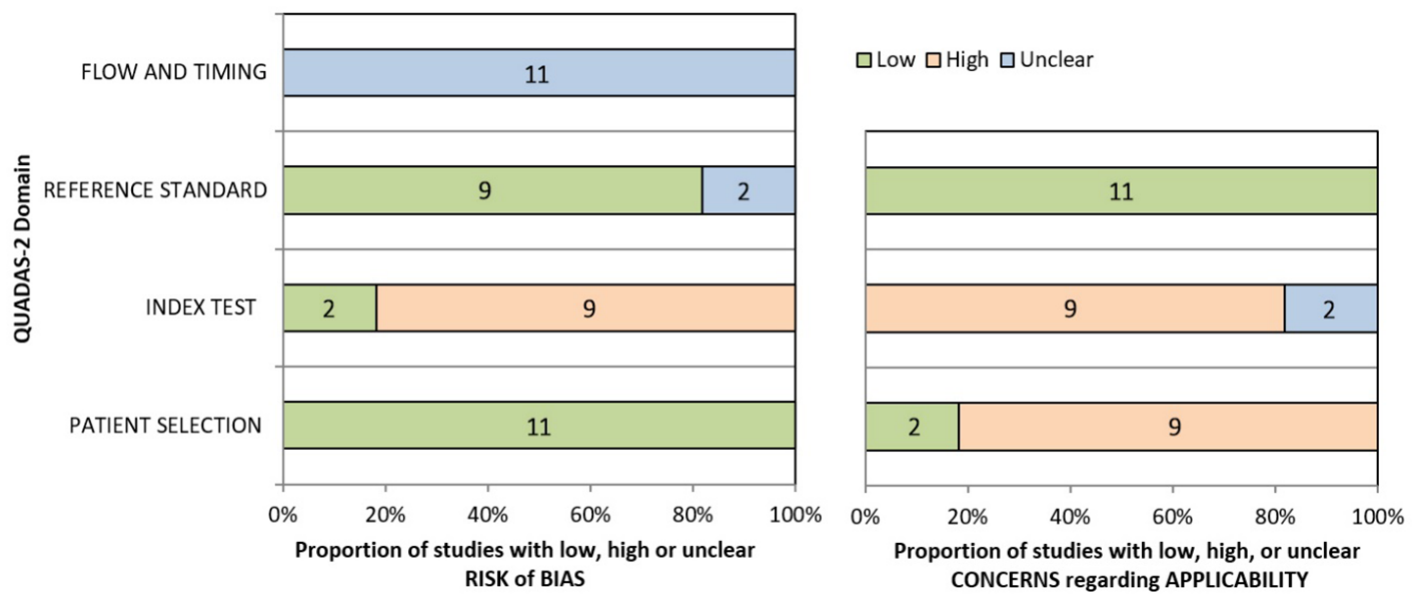
The quality assessment of 11 included studies by QUADAS-2 tool.

**Figure 4:**
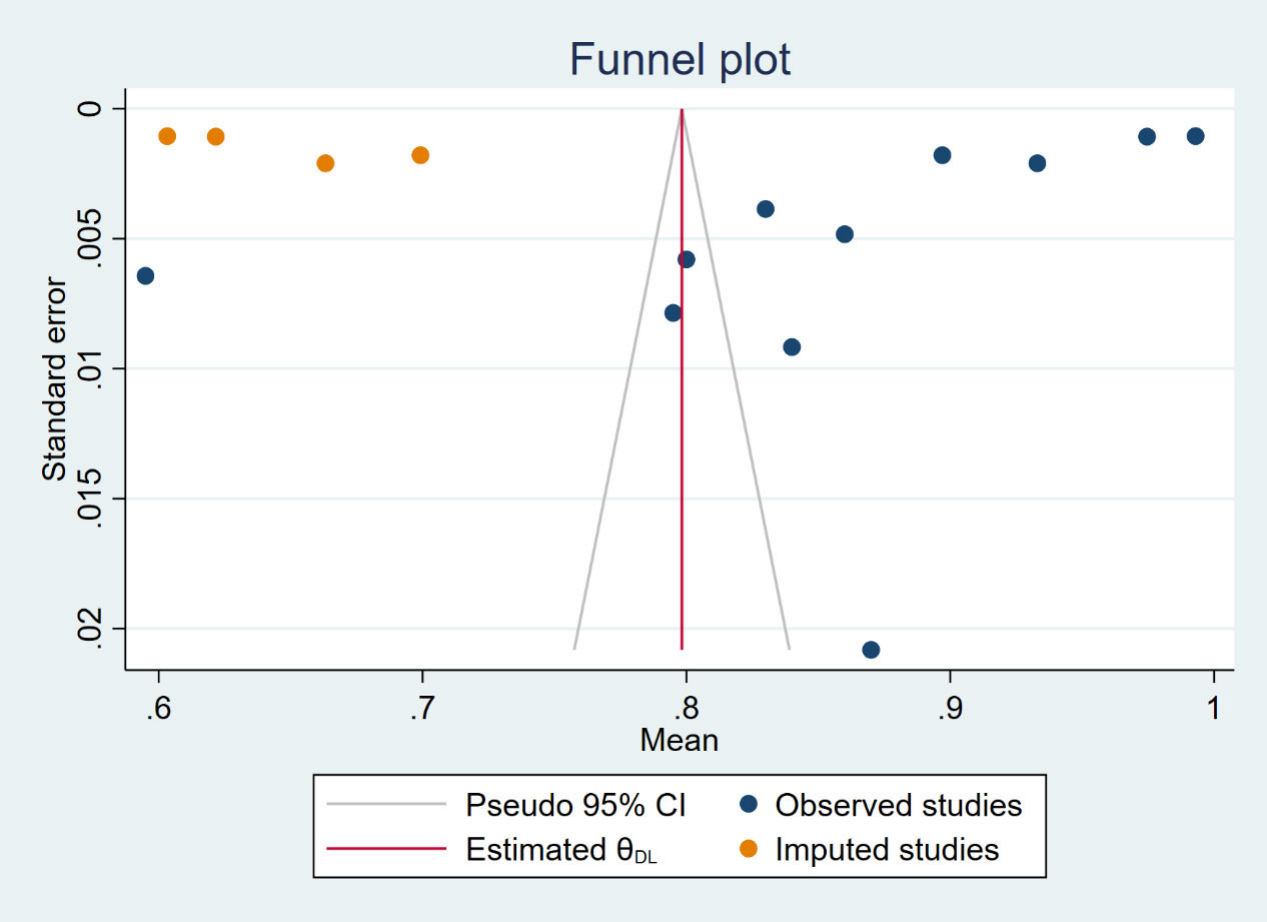
The funnel plot treated by the shear and supplement method.

QUADAS-2 was used to assess the risk of bias in the study, and the results are shown in **Figure 3**. For patient selection, all studies were low risk of bias. However, risk of bias was unclear of flow and timing for all 11 studies. For index test, 9 studies (81.82%) with high risk of bias, 2 studies (18.18%) with low. 9 studies (81.82%) with low and 2 (18.18%) with unclear risk of bias in reference standard. **Table S1** shown individual evaluation of the risk of bias and applicability. For applicability concerns, overall risk is low. Funnel plot (**Figure S1**) and Egger test were used to evaluate whether publication bias existed in the results of the meta-analysis. When publication bias exists, shear and supplement method is used to further analyze whether the results of meta-analysis are stable and reliable (**Figure 4**). In addition, sensitivity analysis was used to evaluate whether the results of the meta-analysis were robust (**Figure S2**).

### Diagnostic accuracy

In these studies, the AUROC, sensitivity and specificity were used to assess the diagnostic performance of models. The categorized data extraction for each study report is shown in **Table 3**. As shown in **Figure 2**, AI models based on ultrasound had the best diagnostic performance, followed by MRI, and CT was the worst. The pooled AUROC of studies based on ultrasound, MRI and CT were 0.94 (95% CI 0.88-1.00), 0.82 (95% CI 0.71-0.93) and 0.82 (95% Cl 0.78-0.86), respectively. In addition, the heterogeneity of all these studies was high, the values of I^2^ reached 99.92%, 99.91% and 92.64% based on ultrasound, MRI and CT. The combined AUROC of all 11 included studies was 0.85 (95%CI 0.81-0.89) and I^2^ was 99.88%.

**Table 3:** Data assessment.

First Author	Sensitivity, %	Specificity, %	Accuracy, %	AUROC	95%CI	Imaging modality
Christiansen-1	0.96	0.867	91.3	0.950	0.897-0.987	Ultrasound
Wang-2	–	0.9	0.9	0.963	0.821-0.945	Ultrasound
Aramendía-3	0.985	0.989	0.9878	0.997	0.862-0.917	Ultrasound
Wang-4	0.69	0.81	0.77	0.83	0.06-0.37	MRI
Li-5	0.9	0.8	0.8	0.87	0.665-0.925	MRI
Liu-6	0.936	0.717	0.828	0.840	0.83-0.96	MRI
Zhuang-7	0.67	0.82	0.76	0.86	0.80-0.99	MRI
Zhang-8	0.9441	0.7885	0.9026	0.9746	0.791-0.943	MRI
Mimura-9	0.762	0.813	_	0.795	0.825-0.94	MRI
Yu-10	0.8	0.75	0.78	0.86	0.716-0.884	CT
Li-11	0.818	0.789	0.805	0.87	0.651-0.912	CT

-, Not report.

## Discussion

Medical imaging is the most effective way to assist clinical diagnosis and analyzing the condition for doctors. Imaging method is important for patients with OC because different images help to determine the feasibility of surgical approach and treatment (4). Our review is the first meta-analysis to evaluate the ability of artificial intelligence to identify benign and malignant ovarian cancer under different imaging modalities.

AI based medical imaging break through the technical barriers of traditional methods which have used in clinical practice, assisting physicians in lesion identification and diagnosis, efficacy assessment, and survival prognosis to improve diagnostic efficiency of doctors (26). Diagnosis of ovarian tumors still requires surgical removal, and the surgeon’s decision making is sometimes challenging in cases where preoperative examination finds atypical. Therefore, if AI can calculate the probability of ovarian cancer based on the results of preoperative examination and predict the final diagnosis, the management level of ovarian cancer will be improved (27). Benign ovarian tumors can avoid unnecessary surgery, and early diagnosis of ovarian cancer can improve the prognosis. In addition, for preoperative diagnosis, patients can receive a more informative probabilistic numerical interpretation (28). Preoperative diagnosis is more accurate and specific in the probability of ovarian tumor management decisions (29). AI extracts features from different types of images differently, and our study shows that the features extracted based on ultrasound images are better overall for the diagnosis of OC (27). Our results are consistent with a previous study which confirmed ultrasound was effective tools to characterize ovarian masses (30).

A total of 11 studies were included in our analysis, of which three were based on ultrasound, six were based on MRI and two based on CT. However, only three of them built deep learning models. This may be due to the fact that deep learning techniques are relatively new and prone to bias. In a recent study, the authors selected the best performed model to extract data for meta-analysis, but in our study, if multiple models were built in a study, we chose the model with the median AUROC value, which may better reflect the overall diagnostic performance of the models in a study. Finally, although most studies divided patients into training and test sets, most of them were monocentric and external validation was particularly important in the study.

However, there are some limitations to our study. First, scanning parameters (including field intensity, contrast agent type, injection velocity, etc.) are not uniform, and the analysis software is different. Then studies that only include Chinese and English literature may have some linguistic bias; In addition, the vast majority of the study’s first authors were from China, as were most of the cases, so there may be some bias. We should also critically consider some methodological issues. Modern information processing techniques to develop radiology report databases can improve report retrieval and help radiologists make diagnoses (31). We need to advocate for Internet networks to identify patient data from all over the world, and large-scale training of AI based on different patient demographics, geographic regions, diseases, and so on. In addition, we highlight the need for a more diverse database of images for rare cancers, including OC.

## Conclusion

AI can play an adjunctive role in identifying benign and malignant ovarian tumors, and the models based on ultrasound has the best diagnostic ability, but due to the limitations of the number and quality of included studies, the above conclusions need to be viewed with caution, and more standardized and prospective studies need to be conducted to confirm them.

In conclusion, AI algorithms show good performance in diagnosing OC through medical imaging. Stricter reporting standards that address specific challenges in AI research could improve future research.

## Author contributions

All authors had full access to all the data in the study and take responsibility for the integrity of the data and the accuracy of the data analysis. QX designed the study. ND, HL and ZL acquired the study data. CY and HW analyzed and interpreted the data. ML wrote the first draft of the manuscript. All authors revised the manuscript and approved it for publication.
