# Supplementary material for: AI diagnostic performance based on multiple imaging modalities for ovarian tumor: A systematic review and meta-analysis
Source: Front Oncol. 2023 Apr 21;13:1133491. doi: 10.3389/fonc.2023.1133491 (PMC10160474; doi:10.3389/fonc.2023.1133491)
Supplement: Supplementary file 1 [file Table_1.docx]

Table S1. Quality assessment
