# Supplementary figures and images for: AI diagnostic performance based on multiple imaging modalities for ovarian tumor: A systematic review and meta-analysis
Source: Front Oncol. 2023 Apr 21;13:1133491. doi: 10.3389/fonc.2023.1133491 (PMC10160474; doi:10.3389/fonc.2023.1133491)

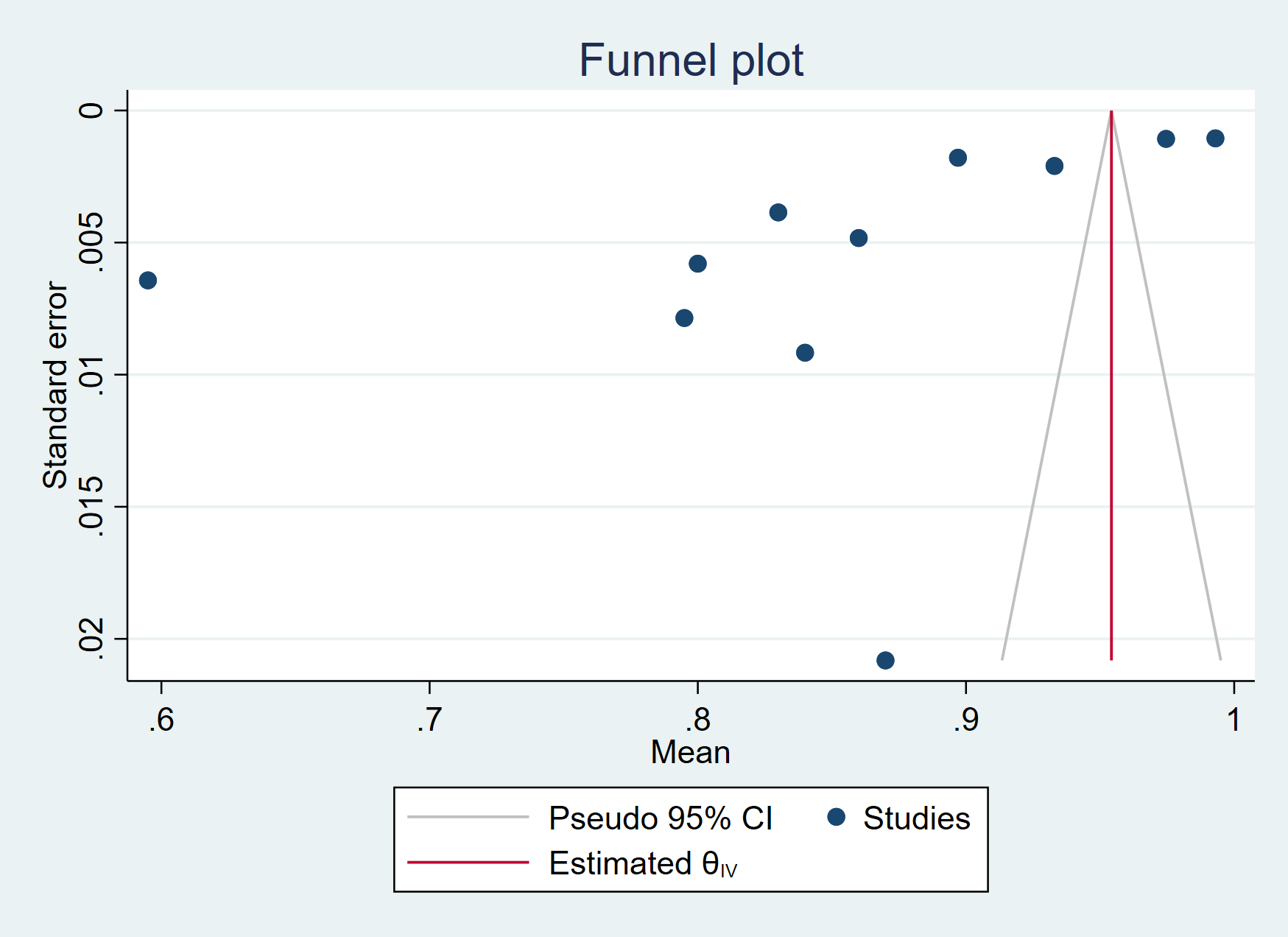

Supplement: Supplementary file 2 [file Image_1.tif]

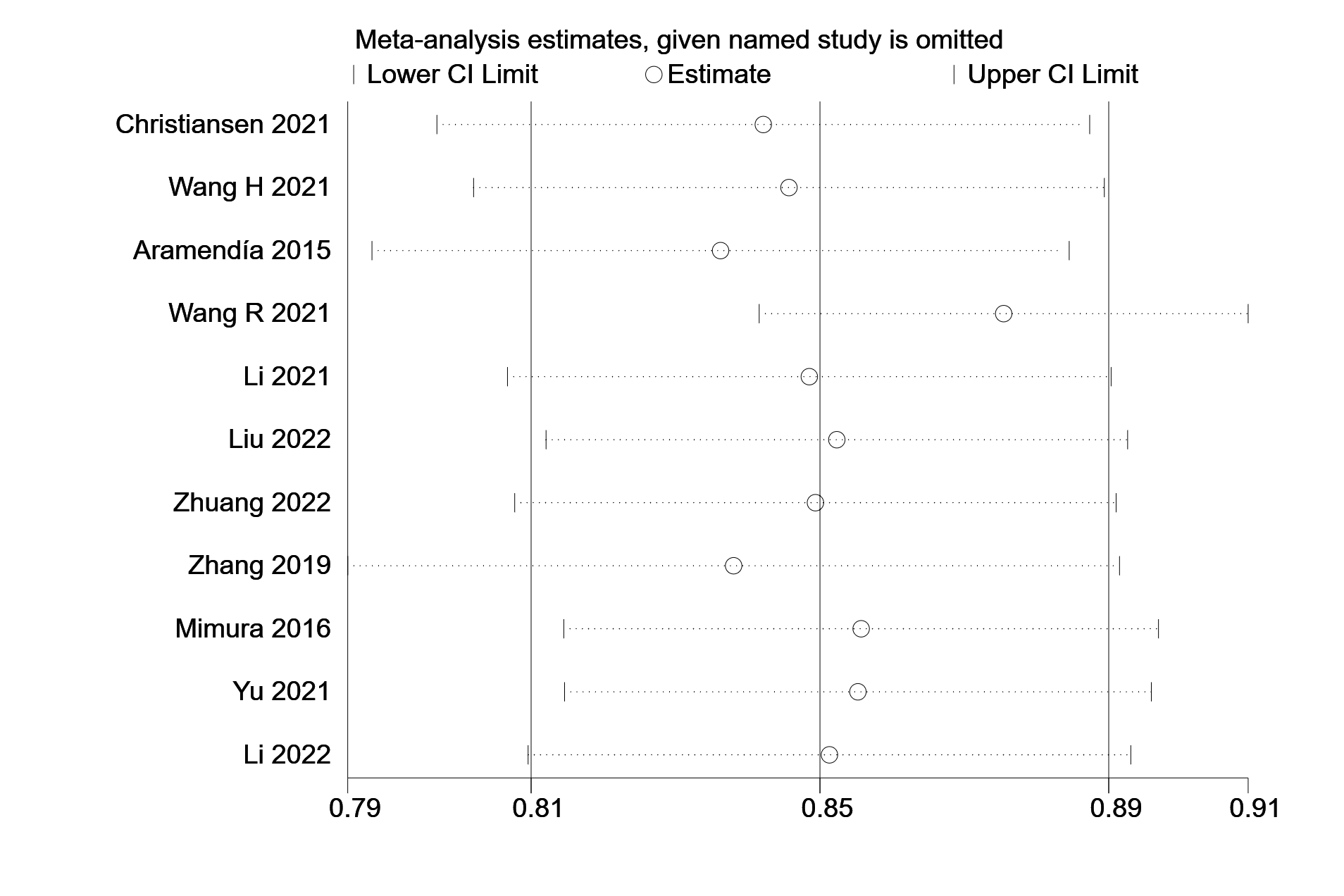

Supplement: Supplementary file 3 [file Image_2.tif]
